# Swiss identity smells like chocolate: Social identity shapes olfactory judgments

**DOI:** 10.1038/srep34979

**Published:** 2016-10-11

**Authors:** Géraldine Coppin, Eva Pool, Sylvain Delplanque, Bastiaan Oud, Christian Margot, David Sander, Jay J. Van Bavel

**Affiliations:** 1 Swiss Center for Affective Sciences, and the Laboratory for the Study of Emotion Elicitation and Expression, University of Geneva, Geneva, Switzerland; 2 The John B. Pierce Laboratory, New Haven, United States; 3 Department of Psychiatry, Yale University, School of Medicine, New Haven, United States; 4 Department of Economics & Laboratory for Social and Neural Systems Research, University of Zürich, Zürich, Switzerland; 5 Firmenich, SA, Geneva, Switzerland; 6 Department of Psychology, New York University, New York, United States

## Abstract

There is extensive evidence that social identities can shape people’s attitudes and behavior, but what about sensory judgments? We examined the possibility that social identity concerns may also shape the judgment of non-social properties—namely, olfactory judgment. In two experiments, we presented Swiss and non-Swiss participants with the odor of chocolate, for which Switzerland is world-famous, and a control odor (popcorn). Swiss participants primed with Swiss identity reported the odor of chocolate (but not popcorn) as more intense than non-Swiss participants (Experiments 1 and 2) and than Swiss participants primed with individual identity or not primed (Experiment 2). The self-reported intensity of chocolate smell tended to increase as identity accessibility increased—but only among Swiss participants (Experiment 1). These results suggest that identity priming can counter-act classic sensory habituation effects, allowing identity-relevant smells to maintain their intensity after repeated presentations. This suggests that social identity dynamically influences sensory judgment. We discuss the potential implications for models of social identity and chemosensory perception.

Classical social psychological theories argue that our construction of social reality is influenced by our membership in groups^1^,^2^,^3^. Indeed, there is extensive evidence that social groups shape our perceptions^4^, judgments^5^, judgments^6^ and memory of ourselves and others^7^. According to Turner and colleagues^8^, social identity may also shape our representations of reality^9^. Therefore, we examined the possibility that social identity concerns may also shape the judgment of non-social properties—namely, olfactory judgment.

Although the study of olfaction has been largely ignored in the field of social identity, there is good reason to believe it might play an important role in intergroup relations. Not only are specific tastes and smells woven into the cultural practices and rituals of many groups (e.g., many ethnic cuisines are associated with distinct foods), people regularly implicate olfaction in the domain of intergroup interactions (e.g., referring to certain out-groups as disgusting^10^. Therefore, understanding the relationship between social identity and olfaction holds the potential promise of understanding the scope of influence exerted by identity as well as providing a new lens to help better understand intergroup relations.

It has traditionally been suggested that the way humans perceive smells is largely innate, hard-wired and predictable, and hence, relatively inflexible^11^. There is, however, some evidence that olfactory judgment may be malleable (for a review, see ref. 12. For instance, olfactory judgment can be influenced by cultural experience^13^,^14^,^15^. Japanese and German people reported differences in the self-reported familiarity, pleasantness, and intensity of common smells^13^. Similarly, people from Switzerland, the United Kingdom, and Singapore reported differences in affective responses (such as disgust, sensuality-desire) to common smells^15^. At a minimum, this suggests that extended cultural exposure can alter olfactory judgment.

It remains unclear whether social identity concerns play any role in shaping olfactory judgment. Cultures differ in countless ways, including exposure to different tastes and smells. People from Singapore might enjoy the pungent Durian fruit due to mere exposure. It is therefore unclear whether familiarity, social identity, or another variable accounts for cross-cultural differences in olfaction.

There is reason to believe that social concerns can influence *visual perception*^16^. For example, identification with a social group—termed social identity^17^ —has been shown to influence the visual judgments of facial stimuli^18^,^19^,^20^,^21^. Social identity may also influence perception judgments of non-social stimuli, making threatening out-group locations seem closer^22^,^23^,^24^ and sound duration appear longer when it is attributed to an in-group^25^. Yet little is known about whether social identity can influence other sensory modalities^9^.

Social identity is the part of people’s self-concept that derives from their knowledge of their membership in a social group together with the value and emotional significance of that membership. Evidence suggests that the accessibility of social identity is dynamic, and varies within cultures^8^,^26^,^27^. During the Olympic games, nationality may be a more salient aspect of identity and influence judgments and behaviors more than usual, despite a person’s cultural background and experiences remaining essentially constant. Since the accessibility of social identities can easily be manipulated independently of exposure to certain smells, it is possible to investigate whether olfactory judgment can be influenced by changes in the perceiver’s social identity. If olfactory judgment was immutable, or at least slow to change, the momentary salience of a perceiver’s social identity should not influence how one reports perceiving an odor. However, if olfactory judgment is sensitive to social identity concerns, priming a perceiver’s social identity might lead to momentary changes in olfactory judgment, increasing sensitivity to identity-relevant smells.

## 

### Current Research

To assess the potential influence of social identity on olfaction, we presented participants with the odor of chocolate, for which Switzerland is world-famous. If olfactory judgment is sensitive to social identity concerns, Swiss identity should influence how an identity-relevant smell like chocolate is judged. Specifically, priming one’s social identity may impact olfactory judgment by increasing the valence or intensity of the smell of chocolate, but not an irrelevant odor (e.g., popcorn).

To see if social identity can influence smell independent of cultural experience, we experimentally primed different social identities. The identity priming procedure was designed to increase the accessibility of participants’ Swiss identity, without simultaneously making it more positive or negative. We speculated that the activation of an important identity would facilitate the judgment of identity-relevant stimuli, if not the valence of the judgment. By affecting the intensity of an olfactory stimulus, identity priming may therefore counteract habituation (i.e., decreasing odor intensity after prolonged exposures^28^). In Experiment 1, we compared a group of Swiss participants primed with their Swiss identity to a group of non-Swiss participants exposed to the same identity priming procedure. The non-Swiss participants were also residents of Switzerland at the time of data collection. This allowed us to isolate the effect of activating *identification* with Switzerland, as opposed to the mere activation of *associations* related to the concept of Swiss identity^29^. In addition, exposing Swiss and non-Swiss participants to the same identity priming procedure allowed us to minimize the likelihood that any differences observed were not due to differences in the primes.

We predicted that Swiss participants primed with their Swiss identity would judge the smell of chocolate as more intense than non-Swiss participants primed with the Swiss identity. We also examined the effect of identity primes on judgments of pleasantness or familiarity. Moreover, we included a go/no-go association task to provide an implicit measure of the accessibility of social identity in Swiss (vs. non-Swiss) participants.

Experiment 2 directly replicated and extended Experiment 1. To examine whether social identity, rather than cultural experience, influences olfactory judgment, we primed different aspects of identity to modify their relative accessibility *within* Swiss participants. This circumvented the ambiguity of cross-cultural comparisons and allowed us to attribute observed differences directly to the accessibility of a social identity. We predicted that Swiss participants primed with the Swiss identity would report the smell of chocolate as more intense than those primed with other identities. To establish discriminatory validity in both experiments, we had participants smell an identity-relevant odorant (chocolate) and an identity-*ir*relevant odorant (popcorn). We expected the Swiss identity priming manipulation to selectively alter olfactory judgments of the identity-relevant smell (chocolate).

## Experiment 1

### Method

#### Participants

Fifty-two University of Geneva students^27^ Swiss, 27 non-Swiss), all reporting a normal sense of smell, took part in this experiment. The non-Swiss participants studied and/or worked in Switzerland, but did not identify with Switzerland. The experiment was advertised in an undergraduate psychology course at the University of Geneva. Individuals could register on a website if they (i) had no olfactory problems, and (ii) they were Swiss and not multi-cultural (Swiss participants group) or were non-Swiss (non-Swiss participants group). The experimenter then contacted them, and checked this information before starting the experiment. Participants were asked not to wear any fragrance during the day of testing. Four participants were excluded because of non-compliance with these requirements: two did not smell the odors as they were instructed to do, one reported contradictory information on his nationality, and one did not complete the questionnaire. As a result, data from 25 Swiss and 25 non-Swiss participants were included in the analyses (mean age = 24 years ± 5 years; 38 females). This research project was started in 2009. In both experiments, an a priori decision was made to determine the sample size. We ran all participants until we reached our a priori target sample size of per condition. The sample size was consistent with norms at that time as well as recommendations from ref. 30. To help ensure reliability, Experiment 2 includes an exact replication of Experiment 1 (see ref. 31 for a discussion about the value of direct replications). All conditions are reported and we have no file drawer studies related to the issue of Swiss identity and olfaction.

Participants received course credit for participating. Participants gave written informed consent, and the study was approved by the ethical committees of the Psychology Department of the University of Geneva. The methods were carried out in accordance with the approved guidelines.

#### Materials

Two odors were used: chocolate and sweet popcorn. These odors were chosen because a pretest had established they were familiar to everyone in our sample, their sources are of similar caloric value and similar macronutrients, and they were differentially associated with Swiss identity. While Switzerland is world-famous for its chocolate, popcorn has no particular association with Switzerland (see Supplementary Materials).

#### Procedure

The experimental session comprised four different phases and was conducted in French language. All materials are freely available online at the Center for Open Science (osf.io/wjkhv).

First, to check for pre-priming differences and establish individual judgment baselines, participants were presented in pseudo-random order with the odors of chocolate and popcorn and were asked to evaluate, on a continuous scale presented on a screen, their pleasantness (from 0 = “*very unpleasant*” to 10 = “*very pleasant*”), familiarity (from 0 = “*not familiar at all*” to 10 = “*very familiar*”) and intensity (from 0 = “*not perceived*” to 10 = “*very strong*”). Participants were then told the labels of the two odors.

Second, participants performed an identity-priming task (adapted from ref. 32). All participants were primed with Swiss identity. Following previous research, we primed different identities by having participants fill in questionnaires. The questionnaires asked participants to list and describe a variety of other attributes (such as typical traits, interests, and foods) associated with Switzerland^32^. Participants were further asked to list and describe three positive and three negative traits of Swiss people. We had participants report both positive and negative traits (intermixed) to avoid selectively enhancing positive or negative valence of the primed identity.

Third, a go/no-go association task^33^ was performed after the priming task. In the go/no-go association task, 16 word stimuli from 4 semantic categories were used: 4 names of Swiss cities (category “*Switzerland*”), 4 names of European cities (category “*Europe*”, the supra category of Switzerland), 4 terms related to the self (category “*self*”) and 4 terms related to others than oneself (category “*others*”). The names of Swiss and European cities were selected to have a similar frequency and number of characters (see Table 1), based on data from a previous pilot test (*N* = 99) that was run on a similar population of students as the one used in the present experiment.

**Figure 1:**
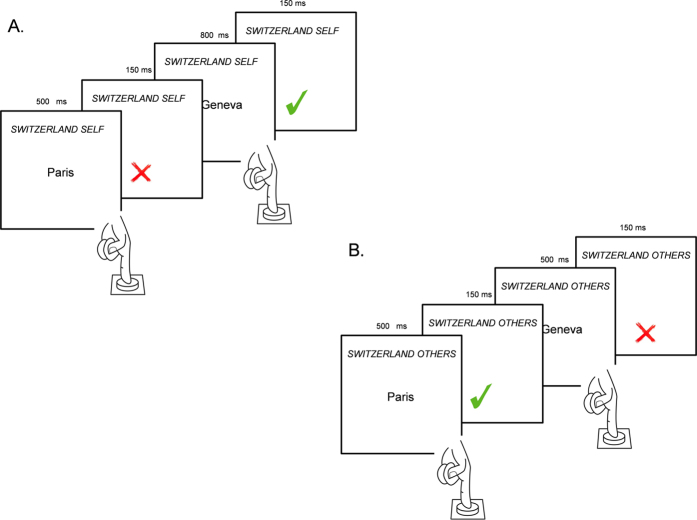
Go/no-go task in Experiment 1. (**A**) Two trials of the Go/no-go block in which participants had to detect whether the words belonged to the target categories “Switzerland” or “Self”: If the stimulus word (Paris) belonged to neither of the two target categories (here, either “Switzerland” or “Self”), the correct response was to not do anything; whereas if the stimulus word (Geneva) belonged to one of the two target categories the correct response was to press ‘A’ on the keyboard. After each response, participants received feedback: Correct responses resulted in a green check mark, whereas incorrect responses resulted in a red X. (**B**) Two trials of the Go/no-go block in which participants had to detect whether the words belonged to the target categories “Switzerland” or “Others”: If the stimulus word (Paris) belonged to one of the two target categories (here, either “Switzerland” or “Other”), the correct response was to press ‘A’ on of the keyboard; where as if the stimulus word (Geneva) belonged to neither of the two target categories the correct response was to not do anything.

The task consisted of two blocks^33^. Each block consisted of 96 trials: 16 training trials and 80 critical trials. In each trial, a *stimulus word* was displayed at the center of the screen. The stimulus word was always a member of one of the four categories (i.e., *Switzerland, Europe, self*, or *others*). The participants’ task was to press the “A” key on the keyboard if they perceived the stimulus word to be a member of at least one of the two *target categories* for that block (*Switzerland* or *self*, for block 1; and *Switzerland* or *others*, for block 2), and to do nothing otherwise (see Fig. 1 for a description of the different trials). During the task, the labels of the target categories were continuously displayed on the top of the screen as a reminder. Non-Swiss European city names were sometimes used as stimulus words, but never used as target categories. Hence, trials in which the stimulus word was a Non-Swiss European city served as distractors. If participants responded after a deadline of 500 or 800 ms, the subsequent trial began. The response deadline was idiosyncratically adapted to the participants’ reaction times and response accuracy: if the response was correct and given with a reaction time shorter than 500 ms, the response deadline for the subsequent trials was 500 ms, otherwise it was 800 ms. After each trial, there was an inter-trial interval of 150 ms during which feedback was displayed (i.e., a green check for correct, and a red cross for incorrect).

Before the two previously described blocks, participants performed a familiarization procedure in which they learned to correctly categorize the words of the four categories. Response accuracy and reaction times were recorded. d’ was computed for each block^33^. First, the proportion of hits (correct go-response for the targets: Switzerland + self, for block 1; and Switzerland + others, for block 2) and false alarms (incorrect go-response for the distractors) were converted to *z*-scores. Subsequently, the difference between the *z-*score values of the hits and false alarms were computed, thus obtaining d’. We then computed a differential score for d’ and for the reaction time by subtracting the scores of the second block (Switzerland + others) from those of the first block (Switzerland + self). This was our primary index of identity accessibility. Higher values on the differential d’ score reflect greater sensitivity when Switzerland and self were targets, compared to when Switzerland and others were targets. Lower values on the differential reaction times index reflect faster responses when Switzerland and self were targets, compared to when Switzerland and others were targets. This was our secondary measure of identity accessibility.

Fourth, the odor rating procedure for pleasantness, familiarity and intensity was repeated for 20 trials for each of the two odors. Odors were presented in pseudo-random order, with the restriction that no more than two consecutive trials included repetitions of the same odor. Participants were also presented with the two odors simultaneously and asked to rate how similar this mixed-odor was to popcorn versus chocolate. This was repeated for 20 trials.

Fifth, participants gave ratings on how much they like the two food items in general (chocolate and popcorn), how often they consume them, and how strongly they associate them with Switzerland on a continuous scale. Participants then completed a social identity questionnaire (adapted from ref. 34) and answered some demographic questions. Participants were then debriefed and dismissed.

#### Statistical analyses

We used Statistica software (Statsoft) for all statistical analyses. The experimental condition (Swiss primed with Swiss identity versus Non-Swiss primed with Swiss identity) was a between-subjects variable while the other factors (odor in the rating task, block in the Go-no Go task) were within-subjects variables.

For each participant and each dimension (pleasantness, familiarity and intensity), we computed the difference scores between the rating before priming and each of the twenty ratings after priming. We then averaged the difference scores across trials. This score ensured that any differences between conditions could be attributed to our priming manipulation rather than pre-existing personal or cultural judgment, as it controls for individual differences in baseline olfactory scores. To ensure the robustness of our results, we also re-ran our analyses with the following dependent measure: (first rating after priming)-(initial rating). However, none of the results obtained this way differed from those reported in the main text. We report results based only on the first measure, since it is more immune to random measurement error, given that it aggregates across all trials.

### Results

The results are similar if we conduct the analysis using separate ANOVAs (as reported in the manuscript) or a three-way MANOVA with the factors social identity, perceptual dimension and odor.

#### Effect of Swiss identity and identity priming on olfactory judgment

**Figure 2:**
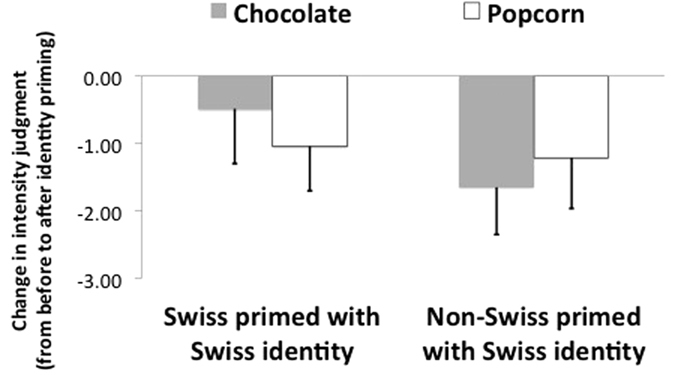
Change in average intensity judgment of chocolate and popcorn odors, from before to after identity priming, for each of the two conditions in Experiment 1. A negative value indicates an intensity judgment decrease after priming. Swiss participants primed with their Swiss identity judged the odor of chocolate as more intense than non-Swiss participants primed with Swiss identity. For popcorn, there was no significant difference in judged intensity across conditions. Intensity was evaluated on a continuous scale. Error bars represent 95% confidence intervals.

Our central hypothesis was that social identity can influence olfactory judgment. Figure 2 illustrates that the effects of Swiss identity on self-reported intensity judgment were specific to the odor of chocolate. A two-way ANOVA with the factors odor (2: chocolate, popcorn) × social identity (2: Swiss primed with Swiss identity, non-Swiss primed with Swiss identity) on intensity difference scores revealed a marginally significant interaction [*F*(1, 48) = 2.90, *p* = 0.095, *η*^*2*^ = 0.057]. More importantly, the main effect of identity on intensity ratings for the chocolate odor was statistically significant, i.e. as predicted, Swiss participants primed with Swiss identity reported the smell of chocolate as more intense (*M* = −0.48, *SD* = 2.08) than non-Swiss participants primed in the same prime condition (*M* = −1.65, *SD* = 1.80) [*t*(48) = −2.11, *p* = 0.040, 95% confidence interval [CI] = [−2.27, −0.06], Hedges’s g_*s*_ = 0.59. The CL (The CL is the “common language effect size statistic”^35^. The idea behind this effect size is to provide an intuitive way to interpret the size of the effect. It measures the magnitude of the difference between two populations by measuring the probability that one person randomly selected from the population 1 scores higher than 1 person randomly selected from the population 2) effect size indicates that there is a 66% likelihood that for a randomly selected pair of individuals the score of a Swiss participant is higher than the score of a non-Swiss participant (see Fig. 2).

Next, we examined whether or not social identity enhances olfactory judgment by buffering against habituation. Specifically, we ran t-tests for single means to investigate whether the averaged intensity difference scores between the rating before priming and all ratings after priming for the odor of chocolate, which was our measure of intensity, were different. Non-Swiss primed with Swiss identity reported chocolate as less intense (*M* = 6.46, *SD* = 1.77) than baseline (*M* = 8.11, *SD* = 1.73) [*t*(24) = 4.58, *p* < 0.001, 95% CI = [0.90, 2.39], g_*av*_ = 0.93, CL = 82%]. In short, participants in this condition reported experiencing olfactory habituation. In contrast, we did not find evidence that Swiss participants primed with Swiss identity reported chocolate as more or less intense than baseline [*t*(24) = 1.36, *p* = 0.255]. This suggests that Swiss identity priming attenuated habituation among Swiss participants.

Two one-way ANOVAs showed that there were no significant effects of condition on chocolate pleasantness (non-Swiss participants: *M* = 0.68, *SD* = 0.27; Swiss participants: *M* = −0.86, *SD* = 1.91) or familiarity ratings (non-Swiss participants: *M* = −0.34, *SD* = 1.88; Swiss participants: *M* = −0.61, *SD* = 1.65) [respectively, *F*_*s*_(1, 48) = 0.93, 2.42, *p*_s_ = 0.341, 0.126], suggesting that the effect of identity priming was specific to intensity. For popcorn, there were also no significant effects of experimental condition on intensity (non-Swiss participants: *M* = −1.04, *SD* = 1.69; Swiss participants: *M* = −1.21, *SD* = 1.93) or pleasantness ratings (non-Swiss participants: *M* = −0.54, *SD* = 2.55; Swiss participants: *M* = 0.01, *SD* = 2.21) [respectively, *F*_*s*_(1, 48) = 0.10, 0.68, *p*_s_ = 0.750, 0.415]. There was, however, an unexpected main effect on familiarity, such that the smell of popcorn was reported more familiar for Swiss (*M* = −0.19, *SD* = 2.10) than non-Swiss participants (*M* = −1.45, *SD* = 1.81) [*t*(48) = −2.26, *p* = 0.003, 95% CI = [−2.37, −0.14], g_*s*_ = 0.63, CL = 68%].

In sum, this suggests that making a particular social identity accessible can selectively enhance the intensity of an odor relevant to the primed identity, without significantly impacting the intensity of an irrelevant odor. Table 2 illustrates all means and standard deviations.

#### The Correlation between Social Identity Accessibility and Olfactory Judgment

To directly assess the relationship between social identity accessibility and olfactory judgment, we analyzed participants’ responses on the Go/no-go association Task^33^. First, we examined the d’ scores (our measure of identity accessibility) among Swiss and non-Swiss participants. A two-way ANOVA with the between-subjects factor social identity (2: Swiss primed with Swiss identity, non-Swiss primed with Swiss identity) and the within-subjects factor block (Switzerland + self; Switzerland + others) on the d’ revealed a marginally significant interaction [*F*(1, 48) = 3.44, *p* = 0.070, *η*^*2*^ = 0.07]. In the first block in which *Switzerland* and *self* were targets, Swiss participants showed a greater sensitivity (*M* = 1, 29, *SD* = 0, 50) than non-Swiss participants (*M* = 0, 65, *SD* = 0, 46) [*t*(48) = −4.72, *p* < 0.001, 95% CI = [−0.91, −0.37], g_*s*_ = 1.31, CL = 83%]. This difference was not present in the second block, in which the target categories were *Switzerland* and *others* [*t*(48) = −0.74, *p* = 0.466]. Taken together, these results are consistent with the notion that Swiss individuals experienced increased accessibility of Switzerland—their group identity—relative to non-Swiss individuals. This is important because participants in both conditions completed the same Swiss priming exercise.

Next, we examined the relationship between olfactory judgment and social identity accessibility using our secondary measure of identity accessibility—the differential index of the reaction times. The difference in this measure between the first and second blocks was marginally correlated with the change in intensity of the smell of chocolate among Swiss individuals primed with Swiss identity [r = −0.39, *p* = 0.056]. We did not find evidence that this was the case for non-Swiss individuals primed with Swiss identity [r = 0.22, *p* = 0.302]. These correlations were significantly different from one another [z = −2.11, *p* = 0.035]. In other words, the intensity of chocolate smell increased as Swiss-identity accessibility increased—but only among Swiss participants.

### Discussion

This experiment provides initial evidence that social identity can influence olfactory judgment. Specifically, we found that priming Swiss people with their national identity counteracted habituation of an identity-relevant chocolate odor. Moreover, these effects tended to be strongest among Swiss participants with the most accessible Swiss identities. Thus the identity accessibility induced by our priming manipulation may underlie the change we observed in olfactory judgments. These effects were not found for the identity-irrelevant popcorn odor. Importantly, priming Swiss identity did not affect a control group of non-Swiss participants: they showed the classic decrease in intensity of the chocolate odor with repeated presentations. This indicates that these effects were not merely due to the priming manipulation or a demand effect associated with the priming manipulation—the results were specific to Swiss participants. The effects of social identity were specific to intensity, not pleasantness or familiarity. This experiment provides the first evidence that social identity can influence olfactory intensity judgments, even in the absence of familiarity differences.

## Experiment 2

Previous research has linked cultural experience to olfaction—a possibility we could not rule out by comparing Swiss versus non-Swiss participants. Therefore, we examined whether social identity, rather than cultural experience, influences olfactory judgment. Specifically, we primed different aspects of identity to modify their relative accessibility *within* a given cultural group—Swiss participants. This circumvents the ambiguity of intergroup comparisons and permits us to attribute observed differences directly to the accessibility of a particular social identity. We predicted that Swiss participants who were primed with the Swiss identity would report the smell of chocolate as more intense than those who were primed with other identities. In contrast, if mere cultural experience was driving the relationship, then Swiss participants would report chocolate as more intense regardless of the accessibility of their social identity.

### Method

#### Participants

Eighty University of Geneva students (inclusion criteria, recruitment and benefits were similar to Experiment 1) took part in this experiment. Two Swiss participants (one primed with his/her Swiss identity, one primed with his/her individual identity) were excluded because they did not comply with the requirements. As a result, data from 58 Swiss and 20 non-Swiss participants were included in the analyses (mean age = 23 years ± 5 years; 66 females).

#### Materials

The sweet popcorn odorant was identical to Experiment 1, whereas the chocolate odorant used in Experiment 1, “chocolate powder”, was similar, but not identical to the one used in Experiment 2 (i.e., “chocolate flavor”) because the production of “chocolate flavor” was discontinued between the conduct of the two experiments.

#### Procedure

The procedure was identical to the one conducted in Experiment 1, except that no go/no-go association task was excluded and two additional groups of participants were added: Swiss participants primed with their individual identity and Swiss participants who were not primed with an identity (see Supplementary Materials).

### Results

#### Effect of Swiss identity and identity priming on olfactory judgment

**Figure 3:**
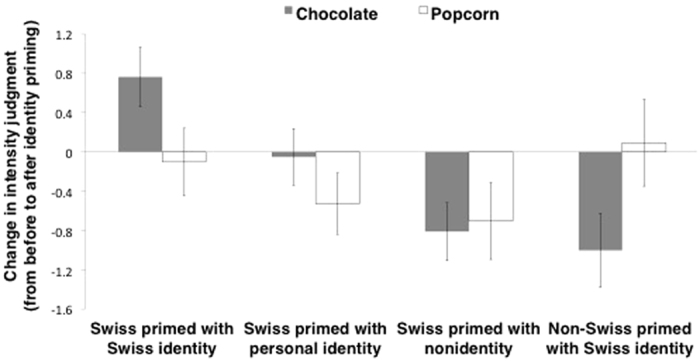
Change in average intensity judgment of chocolate and popcorn odors, from before to after identity priming, for each of the four conditions in Experiment 2. A positive value indicates an intensity judgment increase after priming, while a negative value represents a decrease. Swiss participants primed with their Swiss identity judged the odor of chocolate as more intense than Swiss participants with the individual or nonidentity prime or non-Swiss participants primed with Swiss identity. For popcorn, there was no significant difference in judged intensity across conditions. Intensity was evaluated on a continuous scale. Error bars represent 95% confidence intervals.

Our central hypothesis was that social identity priming would influence olfactory judgment, increasing the intensity of chocolate among Swiss participants primed with their Swiss identity. Figure 3 illustrates that the effects of Swiss identity on intensity were specific to the odor of chocolate. A two-way ANOVA with the factors odor (2: chocolate, popcorn) × social identity condition (4: Swiss primed with Swiss identity, Swiss primed with personal identity, Swiss primed with nonidentity, non-Swiss primed with Swiss identity) on intensity difference scores revealed a significant main effect of the social identity condition [*F*(3, 74) = 2.75, *p* = 0.048, *η*^*2*^ = 0.10] and a significant interaction [*F*(3, 74) = 3.89, *p* = 0.012, *η*^*2*^ = 0.14]. More importantly, the main effect of identity on intensity judgments for the chocolate odor was statistically significant [*F*(3, 74) = 6.32, *p* < 0.001, *η*^*2*^ = 0.20].

Replicating the results of Experiment 1, a *t*-test for independent samples showed that Swiss participants reported the chocolate odor as more intense when primed with Swiss identity (*M* = 0.76, *SD* = 1.33) than Swiss participants who were not primed with an identity (*M* = −0.81, *SD* = 1.32) [*t*(37) = −3.71, *p* < 0.001, 95% CI] = [−2.43, −0.72], g_*s*_ = 1.16, CL = 80%]. Swiss participants primed with Swiss identity also reported the chocolate odor as more intense than non-Swiss participants in the same identity priming treatment (*M* = −1.00, *SD* = 1.68) [*t*(37) = −3.62, *p* < 0.001, 95% CI = [−2.75, −0.78], g_*s*_ = 1.13, CL = 79%]. We found a marginally significant effect of priming Swiss versus individual identity (*M* = −0.05, *SD* = 1.26) in Swiss participants [*t*(36) = −1.93, *p* = 0.061, 95% CI = [−1.66, 0.04], g_*s*_ = 0.61, CL = 67%]. We speculate that the difference between Swiss participants primed with their Swiss versus individual identities might have been slightly weaker than the other contrasts because national and individual identities likely have some overlap among Swiss participants^36^.

**Figure 4:**
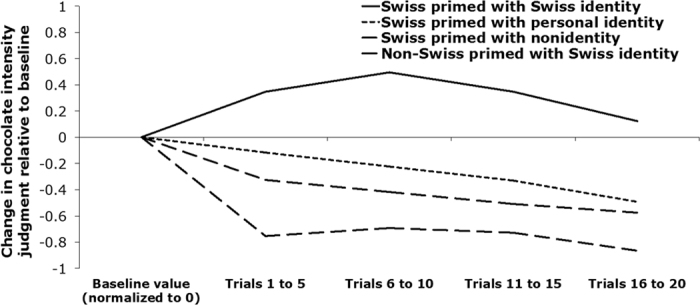
Change in average intensity judgment of the chocolate odor across the 20 trials of Experiment 2, for each of the four experimental conditions, relative to each condition’s pre-priming values. A positive value indicates an increase in judged intensity (after priming-before priming), while a negative value represents a decrease. Note that the drop in judged intensity that one would expect due to habituation is attenuated among Swiss participants primed with Swiss identity. Intensity was evaluated on a continuous scale.

Next, we examined whether or not social identity enhances olfactory judgment by buffering against habituation. Specifically, we ran t-tests for dependent samples to investigate whether the averaged intensity difference scores between the rating before priming and all ratings after priming for the odor of chocolate were different. Swiss participants who were not primed with an identity reported chocolate as less intense (*M* = 7.13, *SD* = 1.63) than baseline (*M* = 7.90, *SD* = 1.10) [*t*(19) = 2.76, *p* = 0.013, 95% confidence interval [CI] = [0.19, 1.36], g_*av*_ = 0.55, CL = 73%] —evidence of habituation. Non-Swiss primed with Swiss identity also reported chocolate as less intense (*M* = 6.53, *SD* = 1.49) than baseline (*M* = 7.49, *SD* = 1.39) [*t*(19) = 2.67, *p* = 0.015, 95% CI = [0.21, 1.70], g_*av*_ = 0.65, CL = 73%]. Thus, participants in both these conditions reported olfactory habituation. However, Swiss participants primed with personal identity did not demonstrate a significant difference (*M* = 6.90, *SD* = 1.35) compared to baseline (*M* = 6.95, *SD* = 1.74) [*t*(19) = −0.18, *p* = 0.859]. Swiss participants primed with Swiss identity actually reported chocolate as *more intense (M* = 7.06, *SD* = 1.58) than baseline (*M* = 6.34, *SD* = 1.78) [*t*(18) = −2.50, *p* = 0.022, 95% CI = [−1.33, −0.12], g_*av*_ = 0.42, CL = 72%] (see Fig. 4). This suggests that Swiss identity priming reversed habituation among Swiss participants.

Replicating the results of Experiment 1, two one-way ANOVAs showed that there were no significant effects of condition on chocolate pleasantness or familiarity ratings [respectively, *F*_s_(3, 74) = 0.23, 0.25, *p*_s_ = 0.872, 0.860], suggesting that the effect of identity priming was specific to intensity. On the contrary, there were no significant effects of condition on intensity, pleasantness, or familiarity judgment for the popcorn odor [respectively, *F*_s_(3, 74) = 0.96, 0.47, 0.66, *p*_s_ = 0.415, 0.706, 0.580], showing that there was no effect of priming for the control odor. This suggests that making a particular social identity accessible can selectively enhance the intensity of odors relevant to the primed identity, without impacting familiarity or pleasantness.

### Discussion

This experiment directly replicates and extends Experiment 1, suggesting that social identity can influence olfactory judgment. Using nearly identical methods, we replicated the finding that priming Swiss people with their Swiss identity judged the smell of chocolate as more intense than non-Swiss people primed with the Swiss identity. Moreover, this effect was specific to an identity-relevant smell, and did not extend to the identity-irrelevant popcorn odor or other dimensions of olfaction, such as pleasantness. Importantly, the results of Experiment 2 show that the effect does not extend to Swiss participants who are primed with their individual identity or receive no identity prime. These results suggest that the effects of Swiss identity on olfactory judgment are due to identity accessibility rather than cultural experience.

### General Discussion

According to Self-Categorization Theory, “All cognition is social cognition from the perspective of the mechanisms of cognition, whether or not it is people or objects that are perceived” (ref. 8, p. 462). Although the term cognition is ordinarily taken to mean information processing that precedes choices and decisions^37^, our research suggests that the influence of self and social concerns might extend to the domain of chemosensory judgment. Priming Swiss participants with their national identity increased the intensity of the identity-relevant chocolate odor, but not an identity-irrelevant popcorn odor. Importantly, priming Swiss identity did not affect non-Swiss participants. The current work offers experimental evidence that social identity can influence olfactory judgment, even in the absence of familiarity differences. Thus, the influence of social identity on cognition may be far more pervasive than originally thought.

We capitalized on the fact that the salience of different identities shifts rapidly to accommodate changes in the immediate social context^8^. Our priming manipulation was designed to enhance identity accessibility without threatening or affirming the identity. Therefore, we expected social identity priming to influence intensity rather than pleasantness or familiarity. As predicted, Swiss identity only appeared to influence olfactory intensity when it was made accessible. Future work should investigate what mediates this relationship. For instance, our data suggest that identity priming may counteract the habituation of an odor after prolonged exposure^28^. Indeed, Swiss participants primed with Swiss identity were the only group to report greater intensity for the chocolate odor relative to baseline. Habituation was delayed relative to the other conditions.

The effect of social identity on olfactory judgment were specific to intensity. This specificity makes it unlikely that simple in-group bias was driving the effect, as it would have likely influenced pleasantness. Although the effect of social identity accessibility was specific to intensity, motivational aspects of one’s identity might be more likely to shape pleasantness judgments. For instance, Canadians who feel threatened by a neighboring country might respond by affirming cultural symbols of their identity, such as reporting greater pleasure when eating a plate of poutine or a dollop of maple syrup (see ref. 38). As such, different aspects of social identity may have an important influence on other dimensions of olfactory judgment.

These results have important implications for the scope of Social Identity Theory^17^ and Self-Categorization Theory^8^ —two highly influential theories in intergroup relations and social psychology. These theories assert that social identities can shape our judgments and perceptions of the self and the social environment^8^. In this vein, social identity appears to influence the processing of social stimuli^39^, estimates of physical distance^22^, and ratings of disgust^10^. The current research extends these theories by showing that the consequences of social identity can influence chemosensory judgment. This suggests that the effect of social identity goes well beyond the domains of self-representations and intergroup behavior^40^ to a host of sensory judgments^9^. Moreover, our results suggest that the influence of self and social concerns on perception is dynamically determined by cues or mental activity that makes certain social identities more or less salient. This goes beyond previous work linking cultural groups and olfaction, but is consistent with extensive work showing that cultural schemes are dynamically constructed^41^. This work is representative of a growing effort to help bridge disparate fields to provide a more complete understanding of human identity and perception.

These experiments advance a growing body of work that discusses the malleability of olfactory judgment^12^. Although the effects we observed are modest in magnitude, they nevertheless suggest that subtle manipulations of social psychological variables *can* impact chemosensory judgment. Thus, cultural effects on olfactory judgment^15^,^42^ may be moderated by the salience and value of perceivers’ social identities^10^. Apart from this work, relatively little research has explored the influence of social concerns on olfaction. At a minimum, this suggests a high degree of flexibility in olfactory judgment.

## Conclusions

The current research demonstrates that olfactory judgment is sensitive to social concerns, which provides speaks to the debate regarding the immutability versus malleability of chemosensory judgment. By suggesting that the influence of identity extends all the way to sensory judgment, our research extends Social Identity Theory^17^and Self-Categorization Theory^8^ —two influential theories in intergroup relations and social psychology. Although these theories have not made any specific claims about sensory-level processes, the results of the current research are consistent with Turner and colleagues’ argument that “all cognition is social cognition”.

## Additional Information

**How to cite this article**: Coppin, G. *et al*. Swiss identity smells like chocolate: Social identity shapes olfactory judgments. *Sci. Rep.* **6**, 34979; doi: 10.1038/srep34979 (2016).

## Supplementary Material

Supplementary Information

## Figures and Tables

**Table 1:** Mean Frequency and Length of the Stimuli used for the Swiss and the European Categories in the Go/no-go Task.

	Stimuli	Frequency	Length
Swiss Category	Bâle (Basel)	9.01%	4
	Fribourg (Fribourg)	6.76%	8
	Genève (Geneva)	27.04%	6
	Neuchatel (Neuchâtel)	10.14%	9
Europe Category	Barcelone (Barcelona)	7.61%	9
	Bruxelles (Brussels)	6.47%	9
	Madrid (Madrid)	10.14%	6
	Paris (Paris)	22.81%	5

**Table 2:** Means by social identity condition and by smell in Experiment 2.

	Swiss primed with Swiss identity	Swiss primed with individual identity	Swiss primed with nonidentity	Non-Swiss primed with Swiss identity
Chocolate-Intensity	7, 47 (SD = 1, 68)	7, 26 (SD = 1, 42)	7, 46 (SD = 1, 76)	6, 82 (SD = 1, 62)
Popcorn-Intensity	7, 17 (SD = 1, 65)	7, 10 (SD = 1, 60)	7, 14 (SD = 1, 94)	7, 02 (SD = 1, 43)
Chocolate-Pleasantness	4, 67 (SD = 2, 41)	5, 06 (SD = 2, 57)	4, 45 (SD = 2, 09)	5, 19 (SD = 2, 15)
Popcorn-Pleasantness	6, 60 (SD = 2, 33)	7, 40 (SD = 2, 11)	5, 66 (SD = 1, 97)	6, 95 (SD = 1, 95)
Chocolate-Familiarity	6, 72 (SD = 2, 30)	6, 76 (SD = 2, 28)	6, 06 (SD = 2, 38)	7, 08 (SD = 1, 82)
Popcorn-Familiarity	7, 95 (SD = 1, 40)	8, 32 (SD = 1, 59)	7, 40 (SD = 1, 63)	8, 06 (SD = 1, 10)
