# Supplementary material for: Swiss identity smells like chocolate: Social identity shapes olfactory judgments
Source: Sci Rep. 2016 Oct 11;6:34979. doi: 10.1038/srep34979 (PMC5057149; doi:10.1038/srep34979)
Supplement: Supplementary Information [file srep34979-s1.docx]

**Swiss identity smells like chocolate:**

**Social identity shapes olfactory judgments**

Géraldine Coppin^1,2,3^, Eva Pool^1^, Sylvain Delplanque^1^, Bastiaan Oud^4^, Christian Margot^5^, David Sander^1^, Jay J. Van Bavel^6^

^1^Swiss Center for Affective Sciences, and the Laboratory for the Study of Emotion Elicitation and Expression, University of Geneva, Geneva, Switzerland

^2^The John B. Pierce Laboratory, New Haven, United States

^3^Department of Psychiatry, Yale University, School of Medicine, New Haven, United States

^4^Department of Economics & Laboratory for Social and Neural Systems Research, University of Zürich, Zürich, Switzerland

^5^Firmenich, SA, Geneva, Switzerland

^6^Department of Psychology, New York University, New York, United States

**Experiment 1**

*Materials*

We thought about using cheese (rather than chocolate) but decided against it for two reasons. First, the perception of a cheese smell is strongly influenced by cognitive factors (43). For instance, thinking of this smell as body odor vs. cheddar cheese drastically impacts ratings. As the first smell presentation occurred without any label, leaving any interpretation of this smell free to each participant, we were afraid that this attribution could compromise the effect of interest. This potential bias was unlikely to happen with the chocolate smell. Second, we wanted to use a control smell which had a similar caloric value, macronutrients content, and familiarity—popcorn fit these three requirements.

To establish that Switzerland is known for its chocolate while popcorn has no particular association with Switzerland, we conducted a pre-test with seventy-one (55 Swiss, 16 non-Swiss) undergraduate psychology students at the University of Geneva. Participants rated the association between ten smell labels (gasoline, chocolate, peanut butter, hamburger, popcorn, donuts, French fries, maple syrup, cheese, and wet clothes) and Switzerland on a 7-point likert scale (from 1 = not at all associated with Switzerland to 7 = very much associated with Switzerland). As shown in Figure 5, the mean association between chocolate and Switzerland (*M* = 5,00, *SD* = 1,62) was higher than the mean association between popcorn and Switzerland (*M* = 2,52, *SD* = 1,33) [*t*(70) = 11.01, *p* < .001, 95 % CI = [2,03, 2,93], g*_av_* = 1.67, CL = 90%].

(Please insert Figure 5 about here)

All odorants used (provided by Firmenich, SA) were diluted in odorless dipropylene glycol to obtain similar evaluations of intensity and presented in cylindrical felt-tip pens (see 44, for further details). The use of these sticks (provided by Burghart, Germany) avoids any olfactory contamination of the environment.

*Results*

*Initial olfactory judgment*

To test whether there were any differences between conditions in familiarity, intensity and pleasantness ratings recorded at the beginning of the study, prior to priming, we ran three separate ANOVAs for these dependent variables. Non-Swiss participants rated the chocolate smell as more pleasant (*M* = 8.36, *SD* = 2.50) than Swiss participants (*M* = 7.00, *SD* = 2.20) [*F*(1,48) = 4.09, *p* =.049, *η^2^* = .08], as well as more familiar (non-Swiss participants: *M* = 8.75, *SD* = 1.78; Swiss participants: *M* = 7.56, *SD* = 1.93) [*F*(1,48) = 5.05, *p* =.029, *η^2^* = .10], although not as more intense (non-Swiss participants: *M* = 8.11, *SD* = 1.73; Swiss participants: *M* = 7.40, *SD* = 1.97) [*F*(1,48) = 1.80, *p* =.185]. To the contrary, no group identity difference was found for the pleasantness, familiarity or intensity of the popcorn [*Fs*(1,48) = 0.02, 0.171, 0.01, *ps* =.889, .681, .915, respectively]. More specifically, averages and standard deviation for the pleasantness, familiarity and intensity of the popcorn for non-Swiss participants were the following: *M* = 5.74, 6.89, 6.81, *SD* = 2.51, 2.63, 1.66, respectively. For Swiss participants, scores were: *M* = 5.30, 6.72, 7.03, *SD* = 2.25, 2.34, 1.72, respectively.

*Self-reported ratings of chocolate and popcorn liking, consumption and associations with Switzerland*

To assess whether the reported general liking of chocolate and popcorn, their general consumption frequency, and their perceived association with Switzerland were different between groups we conducted separate ANOVAs on these three dimensions. We found an increased liking of the popcorn in Swiss individuals (*M* = 7.79, *SD* = 3.36) in comparison to non-Swiss individuals (*M* = 4.78, *SD* = 1.71) [*F*(1, 48) = 16.01, *p* < .001, *η^2^* = .25]. There was no group difference in chocolate liking (non-Swiss participants: *M* = 8.35, *SD* = 1.87; Swiss participants: *M* = 8.28, *SD* = 2.18) [*F*(1, 48) = 0.01, *p* =.905], chocolate consumption (non-Swiss participants: *M* = 7.03, *SD* = 2.22; Swiss participants: *M* = 7.18, *SD* = 2.51) [*F*(1, 48) = 0.05, *p* =.819] or popcorn consumption (non-Swiss participants: *M* = 3.51, *SD* = 2.26; Swiss participants: *M* = 2.93, *SD* = 2.50) [*F*(1, 48) = 0.76, *p* =.388]. This suggests that any effects of social identity on olfaction could not be attributed to reported preferences of consumption.

Importantly, however, we replicated the results of our pilot study, confirming that chocolate (*M* = 8.00, *SD* = 2.48 on a 10-point scale) was more strongly associated with Swiss identity than popcorn (*M* = 1.22, *SD* = 1.88 on the 10-point scale) [*t*(49) = 16.56, *p* < .001, 95% CI = [5.96 , 7.61], g*_av_* = 3.09, CL = 99%]. Thus, chocolate was an identity-relevant smell for Swiss participants, despite the fact that they did not report a stronger preference or greater consumption rates than non-Swiss participants.

**Experiment 2**

*Procedure*

As in Experiment 1, participants performed an identity-priming task (adapted from Ambady, Paik, Steele, Owen-Smith, & Mitchell, 2004). Swiss participants were randomly assigned to one of three priming conditions, in which we primed different aspects of their identity: *Swiss identity* (*N* = 19), *personal identity* (*N* = 19), and *nonidentity* (*N* = 20). A separate sample of *non-Swiss* participants completed the same procedure as participants in the Swiss identity priming condition (*N* = 20). This identity prime was designed to make Swiss identity accessible without threatening or affirming the identity. The questionnaires asked participants to list and describe a variety of other attributes (such as typical traits, interests, and foods) associated either with Switzerland (Swiss identity), themselves (personal identity), or lions (non-identity) (Ambady et al., 2004). Participants were further asked to list and describe three positive and three negative traits of the relevant identity. We asked participants to report both positive and negative traits (intermixed) to avoid selectively enhancing positive or negative valence of the primed identity. Out of the participants primed with Swiss identity, only one mentioned chocolate as a typical food (this participant was non-Swiss), and none mentioned popcorn. This supports our contention that our priming procedure did not activate the concept of chocolate or popcorn in a direct fashion, but rather co-activated it indirectly via its association with Swiss identity.

*Statistical analyses*

The statistical analyses were similar to those conducted in Experiment 1. The experimental condition (Swiss primed with Swiss identity, Swiss primed with personal identity, Swiss primed with non-identity, Non-Swiss primed with Swiss identity) was a between-subjects variable while the other factors were within-subjects variables.

*Results*

*Initial olfactory judgment*

To test whether there were significant differences between conditions in familiarity, intensity, and pleasantness ratings recorded at the beginning of the study, prior to priming, we ran separate one-way ANOVAs for each of this dependent variable. These analyses showed that the chocolate smell was rated at baseline as more pleasant [*F*(1, 77) = 23.30, *p* < .001, *η^2^* = .23] and more familiar [*F*(1, 77) = 27.75, *p* < .001, *η^2^* = .26], but not as more intense [*F*(1, 77) = 0.41, *p* =.522] than the popcorn smell (chocolate’s pleasantness, familiarity and intensity: *M* = 6.12, 7.82, 7.18, *SD* = 2.45, 2.13, 1.60; popcorn’s pleasantness, familiarity and intensity: *M* = 4.61, 5.94, 7.06, *SD* = 2.43, 2.28, 1.70).

*Self-reported ratings of chocolate and popcorn liking, consumption and associations with Switzerland*

To assess possible differences between conditions regarding the measures taken at the end of the study (i.e., for reported general liking of chocolate and popcorn, their general consumption frequency, and their perceived association with Switzerland), we conducted three one-way ANOVAs. Chocolate was rated as more well-liked (*M* = 8.25, *SD* = 2.40 on the 10-point scale) than popcorn (*M* = 6.41, *SD* = 2.59) [*F*(1,77) = 26.45, *p* < .001, *η^2^* = .26]. Further, chocolate was more often consumed (*M* = 7.15, *SD* = 2.82 on the 10-point scale) than popcorn (*M* = 3.17, *SD* = 2.50) [*F*(1,77) = 90.66, *p* < .001, *η^2^* = .54].

Replicating the results of our pilot study and Experiment 1, a separate analysis confirmed that chocolate (*M* = 8.00, *SD* = 2.11 on the 10-point scale) was more strongly associated with Swiss identity than popcorn (*M* = 0.71, *SD* = 0.98 on the 10-point scale), [*t*(77) = 27.83, *p* < .001, 95 % CI = [6.77, 7.82], g*_av_* = 4.70, CL = 100%]. Thus, chocolate was an identity-relevant smell for Swiss participants.

**References**

43. De Araujo, I. E., Rolls, E. T., Velazco, M. I., Margot, C., & Cayeux, I. Cognitive modulation of olfactory processing. *Neuron* **46**, 671-679 (2005).

44. Delplanque, S. et al. Emotional processing of odours: Evidence for a non-linear relation between pleasantness and familiarity evaluations. *Chem. Senses* **33**, 469–479 (2008).


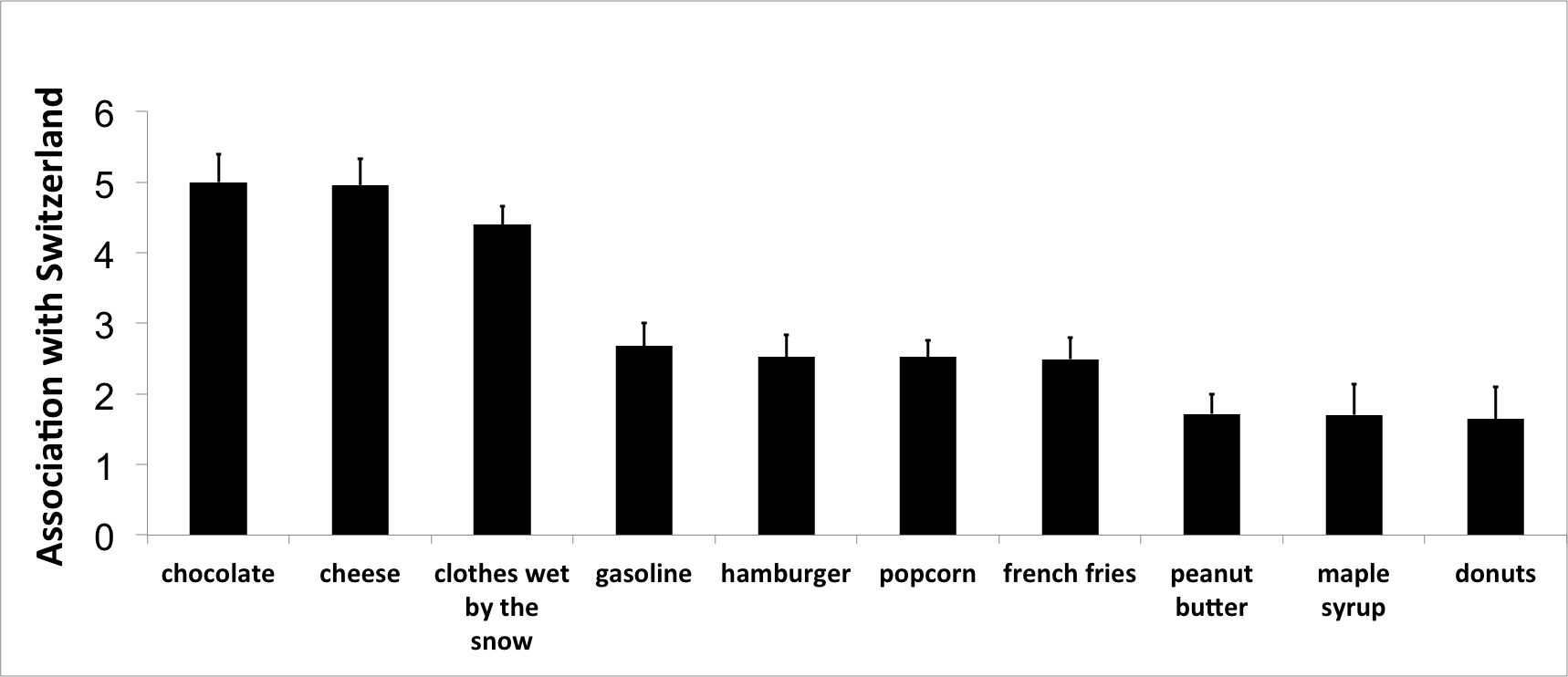


Figure 5. Association found during the pre-test on a 7-point likert scale (from “not at all” to “very much”) between Switzerland and the smells of chocolate, cheese, clothes wet by the snow, gasoline, hamburger, popcorn, French fries, peanut butter, maple syrup and donuts. The smell of chocolate was significantly more associated with Switzerland than the smell of popcorn. Error bars represent the 95% confidence intervals.
